# Renal function dynamics in COVID-19: exploring biomarker interactions with D-dimer and C-reactive proteins

**DOI:** 10.1042/BSR20254002

**Published:** 2026-04-22

**Authors:** Ishita Saha, Anirban Sinha, Anup Kumar Sadhu, Rabindra Nath Das, Satadru Ghosh, Priyajit Banerjee, Oly Banerjee, Shampa Sarkar Biswas, Sandip Mukherjee, Palash Kumar Pal

**Affiliations:** 1 Department of Physiology, Medical College and Hospital, Kolkata 700073, West Bengal, India; 2 Department of Endocrinology, Medical College and Hospital, Kolkata 700073, West Bengal, India; 3 Consultant Radiologist, EKO Diagnostic, Medical College and Hospital, Kolkata 700073, West Bengal, India; 4 Department of Statistics, The University of Burdwan, Golapbag, Burdwan 713104, West Bengal, India; 5 Laboratory of Drug Discovery and Protein Engineering, School of Life Sciences, Swami Vivekananda University, Barrackpore 700121, West Bengal, India; 6 Department of Medical Laboratory Technology, School of Allied Health Sciences, Swami Vivekananda University, Barrackpore 700121, West Bengal, India; 7 Department of Life Sciences, Presidency University, Kolkata 700073, West Bengal, India; 8 Department of Physiology, Serampore College, Hooghly 712201, West Bengal, India; 9 Eco-toxicology and Environmental Endocrinology Laboratory, School of Life Sciences, Swami Vivekananda University, Barrackpore 700121, West Bengal, India

**Keywords:** Blood urea nitrogen, C-reactive protein, COVID-19, Creatinine, D-dimer, Renal function

## Abstract

COVID-19, caused by the SARS-CoV-2 virus, is mainly recognized for its respiratory manifestations. However, growing evidence regarding the widespread expression of ACE2 and TMPRSS2 receptors on diverse extrapulmonary sites, particularly in renal tubular epithelial cells, suggests susceptibility of other organ systems, including the kidneys, to such conditions as acute kidney injury (AKI). In the present retrospective study, we explored the interrelationship between disease severity and renal function abnormalities by analyzing key biochemical parameters: blood urea nitrogen (BUN), serum creatinine (Cr), the BUN/Cr ratio, and estimated glomerular filtration rate (eGFR). Using descriptive statistics and joint generalized linear models, we examined both the mean and variance components of these markers alongside inflammatory indicators such as C-reactive protein (CRP) and D-dimer. Our findings revealed a significant positive correlation between serum urea levels and both CRP and D-dimer concentrations, suggesting that elevated urea may reflect heightened inflammatory activity. Additionally, eGFR showed a positive association with CRP, indicating potential renal involvement in systemic inflammation. Our *in silico* studies supported such observations, as genes responsible for CRP and D-dimer elevation were found to be common in AKI-associated pathways, particularly IL-6/JAK-STAT, NF-κB, HIF-1, and complement pathways, ultimately causing renal microthrombosis, tubular necrosis, and fibrotic remodeling. Notably, serum Cr revealed no significant association with CRP or D-dimer, possibly due to its lower sensitivity in early renal dysfunction. Although the study is limited by a relatively small sample size and lacks longitudinal data, it underscores the importance of monitoring renal function parameters in COVID-19 patients as potential markers of disease progression.

## Introduction

On January 30, 2020, the World Health Organization (WHO) declared COVID-19 a global public health emergency of international concern. Since then, the pandemic has inflicted catastrophic consequences worldwide, claiming over 7 million lives and debilitating many more as of April 19, 2024 [1,2]. COVID-19 is caused by severe acute respiratory syndrome coronavirus 2 (SARS-CoV-2), a zoonotic strain of bat coronavirus. The virus spreads primarily through airborne droplets sprayed during coughing or sneezing by infected individuals and can also be conveyed indirectly via contaminated materials (fomites) [3,4].

Among the four structural proteins of SARS-CoV-2, the spike (S) protein exerts a key role in human-to-human transmission [5]. This protein binds to the angiotensin-converting enzyme 2 (ACE2) receptor on human cell membranes, facilitating viral entry. Additionally, the transmembrane serine protease TMPRSS2 on host cell membranes primes the spike protein by cleaving it into S1 and S2 subunits [6]. The receptor-binding domain on the S1 subunit binds to ACE2, while the S2 subunit mediates the fusion of viral and host cell membranes, enabling nucleic acid delivery, replication, and proliferation within the host cells [7–9].

Initially presenting as clusters of pneumonia cases, COVID-19 was soon recognized as a multi-systemic disease due to the virus’s tropism for various extrapulmonary sites. The co-expression of ACE2 and TMPRSS2 in vital tissues/organs, including the hepatic, cardiac, renal, nervous, and gastrointestinal tissues—underpins this pan-systemic affinity [10].

Kidneys exhibit notably high ACE2 expression levels, nearly 100 times higher than in the lungs, particularly along the apical brush border of proximal tubular epithelial cells [11]. Urine extracellular vesicle studies indicate consistent ACE2 receptor expression in proximal tubular cells (PTC), irrespective of SARS-CoV-2 infection status or gender [12]. While TMPRSS2 expression is elevated in SARS-CoV-2-infected male patients compared with females or healthy individuals, the co-localization of ACE2 and serine proteases in renal epithelium renders this tissue vulnerable to viral infection [13,14]. Single-cell RNA sequencing data further corroborates this, showing 4% ACE2 positivity in PTC epithelial cells compared with 1% in type II alveolar cells of the lungs, designating renal tubular epithelial cells as high-risk targets [15,16].

Global studies have documented renal function impairment in severe COVID-19 cases, with the incidence of acute kidney injury (AKI) ranging from 1% to 46%, though data heterogeneity exists [17]. AKI in COVID-19 manifests as elevated serum creatinine (Cr) and blood urea nitrogen (BUN) levels, alongside a decline in glomerular filtration rate (GFR). Despite widespread recognition of these dysregulated parameters, the extent of their association with clinical outcomes remains unclear [18].

To address this gap, the present hospital-based retrospective study examines the relationship between COVID-19 disease severity and renal function abnormalities during the acute phase of illness. We employed RT-PCR Ct values as an inverse indicator of viral load, with higher Ct values corresponding to lower viral loads [19]. Disease severity was classified based on WHO guidelines, considering respiratory rates (>30 breaths/min), arterial oxygen saturation levels (<93% at room air), and severe respiratory distress. Additionally, D-dimer and C-reactive protein (CRP) levels were utilized as markers of hyper-inflammation and adverse outcomes.

## Methodology

### Study design and participants

This retrospective investigation comprised hospitalized symptomatic COVID-19 patients. Conducted between May 2020 and November 2021, the study analyzed data from the bed head tickets of 150 adult patients with COVID-19 positive results. Participants were aged 18 yr or older, with no gender-based restrictions.

Medical records of individuals under 18 yr of age and pregnant women were excluded during the data collection phase. Additionally, patients undergoing maintenance dialysis or those with a history of chronic kidney disease (CKD) were not included in the study. Since this was a retrospective study, informed consent was not obtained; however, all patient identities were anonymized to ensure confidentiality.

### Study variables

In the present investigation, data were primarily collected on the demographic and clinical features of the participants, including sex, age, and patient itinerary (encompassing the duration of hospitalization in general wards and ICUs). Details regarding COVID-19 diagnosis, such as diagnostic test results and cycle threshold (Ct) values, were also obtained. Key variables analyzed included serum urea (an estimate of BUN), serum Cr, and the serum urea-to-creatinine ratio (BUN/Cr). Additionally, serum Cr levels were used to determine the estimated glomerular filtration rate (eGFR), while accounting for patients’ age and gender. Acute-phase reactants, such as CRP and D-dimer, were further assessed as part of the study. Additionally, SpO_2_ levels and respiratory rates serve as key indicators of disease severity based on WHO criteria.

### Data collection

The biochemical test results in the present study were documented either at the time of admission or within the initial 24 h of patients’ stay in the COVID-19 wards. During this period, patients primarily received treatments aimed at alleviating symptoms, which included antibiotic therapies such as azithromycin (500 mg OD for 5 days), doxycycline (100 mg BD for 7 days), and/or a combination of amoxicillin-clavulanic acid (625 mg thrice daily for 5 days). In certain cases, ivermectin (12 mg OD for 5 days) was administered alongside doxycycline. A small proportion of patients were given anticoagulant therapy, specifically heparin of lesser molecular weight (40 mg subcutaneously once daily), within the first 24 h of hospitalization. Notably, no patients received antiviral drugs (e.g., remdesivir or ritonavir), glucocorticoids, or other medications known to potentially influence renal function during this timeframe.

### *In silico* studies

Different databases, like GeneCards and OMIM database, were utilized to list down various genes responsible for elevating the common biomarkers of COVID-19 along with the various genes associated with the AKI pathways. To identify the genes from GeneCards, genes with high threshold values—‘relevance scores’—were taken into account to assure greater binding with the phenotype. Clinically documented data sets are based in the OMIM database—thus, a separate gene list was generated that mostly had documented or confirmed genes with the pathophysiology of the disease AKI, and genes reported for COVID-19 directly linked to clinically recognized biomarkers were found out. Then these three sectors were overlapped to find the composite gene list and selected and further analyzed through KEGG pathway analysis at ShinyGO 0.82.

### Statistical analysis

Genstat (version 12), SPSS (version 26), and GraphPad Prism (version 8.0) were used for statistical analysis, considering *P* <0.05 as the threshold of statistical significance. Collected enormous data were summed up using measures such as mean, standard deviation, median, and interquartile range (IQR). To explore the relationships between acute-phase reactants (C-reactive protein and D-dimer) and variables like respiratory rate, oxygen saturation, and renal function parameters, the joint generalized linear model (JGLM) was employed. This approach facilitated simultaneous modeling of the data’s mean and variance components, offering comprehensive insights into the interactions among the biochemical markers.

### JGLMs for log-normal distribution

For the positive response *Y*_*i*_ (= CRP or D-dimer) with E (Y*_i_ *= CRP or D-dimer) = μ_*i*_ (mean) and Variance (*Y_i_ *= CRP or D-dimer) = $\sigma_{i}^{2}\mu_{\text{i}}^{2}=\sigma_{i}^{2}$ *V*(*μ_i_*) say, where $\sigma_{i}^{2}$’s are dispersion parameters and *V* (·) reveals the variance function. Generally, log transformation *Z_i_ *= log (*Y*_*i*_ = CRP or D-dimer) is adopted to stabilize the variance Var (Z_*i*_) ≈ $\sigma_{i}^{2}$, but the variance may not always be stabilized [15]. For developing a CRP or D-dimer improved model, JGLMs for the mean and dispersion are considered. For the response CRP or D-dimer, assuming log-normal distribution, JGL mean and dispersion models [with Z*_i_ *= log (Y_*i*_ *=* CPK)] are as follows:

$$\text{E}({\text{Z}}_{\text{i}})={\text{μ}}_{\text{zi}}\;\;\text{and }\;\text{Var}({\text{Z}}_{\text{i}})=\sigma_{\text{zi}}^{2},$$

$${\text{μ}}_{\text{zi}}={\text{x}}_{\text{i}}^{\text{t}}\;\beta \;\text{ and }\;\log(\sigma_{\text{zi}}^{2})={\text{g}}_{\text{i}}^{\text{t}}\;\gamma ,$$

where, x*_i_^t^* and g*_i_^t^* are the explanatory factors/variables, vectors of CRP or D-dimer associated with the mean regression coefficients β and dispersion regression coefficients γ, respectively. The final models for CRP and D-dimer were selected based on the smallest Akaike information criterion (AIC) values within each class, ensuring the minimization of both squared error loss and predictive additive errors.

## Results

### Demographic and laboratory characteristics

Demographic and laboratory features of the subjects are depicted in Table 1. The study included 150 individuals with a mean age of 56.72 ± 17.48 yr (95% CI: 53.89–59.55) and a median age of 58.5 yr with an IQR of 45.5–70.0 yr. The duration of hospital stays averaged 9.45 ± 6.19 days (CI: 8.45–10.46), with a median of 8.0 days (IQR: 5.0–12.0). D-dimer levels, which normally range between 0.1 and 0.5 μg/ml, were markedly elevated with a mean of 6.61 ± 16.46 μg/ml (CI: 3.94–9.27) and a median of 2.06 μg/ml (IQR: 0.65–4.17). Similarly, CRP levels, typically <0.3 mg/dl, showed a mean of 48.37 ± 38.97 mg/dl (CI: 42.06–54.68), with a median of 38.3 mg/dl (IQR: 10.46–83.75), indicating significant inflammation.

**Table 1:** Demographic and laboratory characteristics of study participants

Parameters	*n*	Mean ± SD	CI (95%)	Median	IQR
Age (years)	150	56.72 ± 17.48	53.89–59.55	58.50	45.5–70.0
Duration of hospital stay (days)	150	9.453 ± 6.19	8.45–10.46	8.00	5.0–12.0
D-dimer	150	6.606 ± 16.46	3.94–9.27	2.06	0.65–4.17
CRP (<0.3 mg/dl)	150	48.37 ± 38.97	42.06–54.68	38.30	10.46–83.75
Ct values	150	24.91 ± 5.31	24.05–25.77	25.0	20.52–28.69
Urea (16 to 48.5 mg/dl)	150	87.90 ± 85.86	74.00–101.80	56.0	31.0–124.75
Creatinine (1.3 mg/dl)	150	2.17 ± 2.24	1.81–2.54	1.20	0.90–2.48
eGFR (ml/ min/1.73 m^2^)	150	59.90 ± 35.21	54.20–65.60	64.0	23.25–87.75
BUN/Cr	150	44.49 ± 24.01	40.60–48.37	42.0	26.36–56.20
SpO_2_ (94%)	150	91.25 ± 8.59	89.86–92.64	93.0	90.0–96.0
Respiratory rate (12–18/min)	150	24.19 ± 6.17	23.19–25.19	25.0	19.0–29.0

The normal reference ranges for biochemical and physiological parameters are provided in parentheses.

CRP: C-reactive protein; Ct value: cycle threshold value, eGFR: estimated glomerular filtration rate, SpO_2_: oxygen saturation.

The mean Ct value was 24.91 ± 5.31 (CI: 24.05–25.77), with a median of 25.0 (IQR: 20.52–28.69). Urea levels, normally between 16 and 48.5 mg/dl, were elevated with a mean of 87.90 ± 85.86 mg/dl (CI: 74.00–101.80) and a median of 56.0 mg/dl (IQR: 31.0–124.75). Serum Cr averaged 2.17 ± 2.24 mg/dl (CI: 1.81–2.54), with a median of 1.20 mg/dl (IQR: 0.90–2.48), suggesting renal involvement. The eGFR was 59.90 ± 35.21 ml/min/1.73 m^2^ (CI: 54.20–65.60), with a median of 64.0 (IQR: 23.25–87.75). The BUN/Cr ratio was 44.49 ± 24.01 (CI: 40.60–48.37), with a median of 42.0 (IQR: 26.36–56.20). Oxygen saturation (SpO_2_) was slightly below normal, with a mean of 91.25 ± 8.59% (CI: 89.86–92.64) and a median of 93.0% (IQR: 90.0–96.0). The respiratory rate, normally 12–18 breaths per minute, was elevated with a mean of 24.19 ± 6.17/min (CI: 23.19–25.19) and a median of 25.0/min (IQR: 19.0–29.0), indicating respiratory distress in many patients.

### Response of D-dimer

The final D-dimer model was selected on the basis of the lowest AIC value within each distributional class, optimizing both squared error loss and predicted additive errors. According to the AIC criterion, the gamma fit (AIC = 590.009) outperformed the log-normal fit (AIC = 650.240), indicating a superior model under the framework of JGLMs (Table 2).

**Table 2:** Results for mean and dispersion models for D-dimer from log-normal and gamma fit

Model	Covariates	Log-normal FIT				Gamma fit			
		Estimate	S.E.	*t*	*P*-value	Estimate	S.E.	*t*	*P*-value
Mean	Constant	−4.0919	2.2517	−1.817	0.0715	0.1463	1.8210	0.080	0.9363
	CRP	−0.0072	0.0266	−0.269	0.7883	−0.0424	0.0214	−1.986	0.0491
	Ct	0.0925	0.0218	4.237	<0.0001	0.0877	0.0186	4.704	<0.0001
	eGFR	0.0191	0.0117	1.638	0.1038	0.0037	0.0100	0.369	0.7127
	SpO_2_	−0.0025	0.0166	−0.152	0.8794	−0.0233	0.0123	−1.895	0.0603
	Dura	−0.0464	0.0102	−4.530	<0.0001	−0.0264	0.0084	−3.134	0.0021
	Urea	0.0077	0.0027	2.827	0.0054	0.0132	0.0023	5.866	<0.0001
	CRP*urea	−0.0001	0.0001	−3.636	0.0004	−0.0001	0.0001	−5.436	<0.0001
	Cr	0.1965	0.1605	1.224	0.2231	0.1417	0.1462	0.969	0.3343
	CRP*Cr	0.0071	0.0016	4.443	<0.0001	0.0082	0.0015	5.619	<0.0001
	CRP*SpO_2_	0.0005	0.0002	2.444	0.0158	0.0006	0.0002	3.681	0.0003
	Rrate	0.2400	0.0450	5.331	<0.0001	0.0868	0.0410	2.119	0.0360
	CRP*Rrate	−0.0021	0.0004	−4.813	<0.0001	−0.0008	0.0004	−2.000	0.0475
	Ct*Cr	−0.0261	0.0079	−3.323	0.0011	−0.0299	0.0066	−4.535	<0.0001
	eGFR*Rrate	−0.0016	0.0005	−3.440	0.0008	−0.0007	0.0004	−1.606	0.1107
	Gen 2	−0.3186	0.1995	−1.597	0.1126	−0.2397	0.1725	−1.390	0.1669
	eGFR*Cr	−0.0085	0.0083	−1.021	0.3091	−0.0098	0.0065	−1.517	0.1316
	CRP*eGFR	0.0002	0.0001	3.025	0.0030	0.0002	0.0001	3.464	0.0007
Dispersion	Constant	−13.48	3.89	−3.47	0.0007	−12.49	4.26	−2.93	0.0040
	Urea	−0.0115	0.0085	−1.35	0.1793	−0.0121	0.0096	−1.26	0.2099
	Rrate	0.2768	0.0613	4.52	<0.0001	0.2632	0.0667	3.95	0.0001
	Ct	0.211	0.105	2.01	0.0465	0.194	0.115	1.69	0.0934
	Gen 2	1.77	1.45	1.22	0.2246	2.50	1.59	1.57	0.1188
	Ct*Gen 2	−0.1058	0.0491	−2.15	0.0334	−0.1737	0.0540	−3.22	0.0016
	eGFR	0.0251	0.0182	1.38	0.1699	0.0028	0.0198	0.14	0.8889
	SpO_2_	0.0549	0.0194	2.83	0.0054	0.0580	0.0215	2.69	0.0081
	Dura	−0.425	0.119	−3.57	0.0005	−0.386	0.118	−3.26	0.0014
	Ct*Dura	0.0145	0.0044	3.32	0.0012	0.0131	0.0044	2.96	0.0036
	CRP	0.0635	0.0137	4.63	<0.0001	0.0682	0.0151	4.52	<0.0001
	Urea*CRP	−0.0001	0.0001	−1.68	0.0953	−0.0002	0.0001	−2.87	0.0048
	Rrate*CRP	−0.0024	0.0006	−4.10	<0.0001	−0.0024	0.0006	−3.68	0.0003
	Urea*eGFR	−0.0002	0.0001	−2.21	0.0288	−0.0001	0.0001	−1.39	0.1669
	Rrate*eGFR	−0.0015	0.0007	−2.31	0.0224	−0.0012	0.0007	−1.59	0.1142
	eGFR*Gen 2	0.0126	0.0073	1.72	0.0878	0.0234	0.0081	2.90	0.0044
	age	0.0981	0.0405	2.42	0.0169	0.0739	0.0428	1.73	0.0860
	Ct*age	−0.0049	0.0017	−2.78	0.0062	−0.0038	0.0019	−2.02	0.0454
	Urea*age	0.0002	0.0001	1.53	0.1284	0.0002	0.0001	1.70	0.0915
AIC	650.240					590.009			

CRP: C−reactive protein; Ct value: cycle threshold value, eGFR: estimated glomerular filtration rate, SpO_2_: oxygen saturation, Dura: duration of hospital stay, Cr: creatinine, Rrate: respiratory rate, Gen: gender, AIC: Akaike Information Criterion.

**Figure 1:**
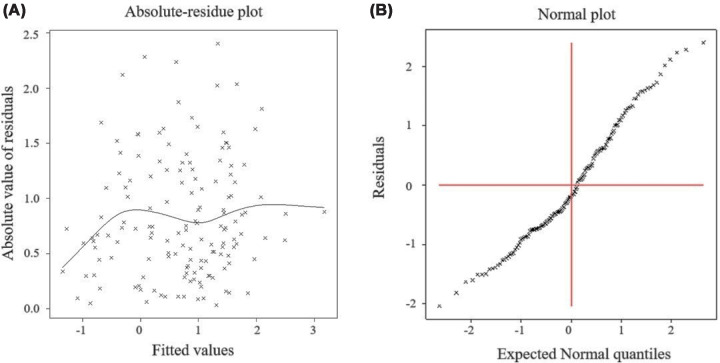
Diagnostic plots for joint log-normal-fitted models of D-dimer For joint log-normal-fitted models of D-dimer, (**A**) the absolute residuals plot with the fitted values, and (**B**) the normal probability plot for the mean model.

Figure 1A displays the absolute residuals plotted against the fitted values for the log-normal D-dimer model. The residual curve appears approximately flat, suggesting homoscedasticity and a constant variance across the range of fitted values. Figure 1B presents the normal probability plot for the fitted mean model, which shows no evidence of lack of fit. Together, these diagnostic plots confirm the adequacy of the log-normal model, which has therefore been accepted as a rational estimate of the underlying D-dimer distribution in the present study.

Analysis of the mean D-dimer levels revealed a statistically significant positive association with serum urea (P <0.0001) and a negative association with C-reactive protein (CRP) (*P* = 0.0491). Notably, the joint interaction effect (JIE) of CRP and urea (CRP × urea) was negatively associated with mean D-dimer levels (P <0.0001), suggesting that D-dimer concentrations tend to rise as the combined influence of CRP and urea diminishes. While serum Cr alone showed no significant association with mean D-dimer (*P* = 0.3343), its interaction with CRP (CRP × Cr) was positively associated (*P* <0.0001), indicating that elevated D-dimer levels correspond with increased CRP × Cr interaction.

Similarly, the joint interaction of CRP and oxygen saturation (CRP × SpO_2_) was positively associated with mean D-dimer (*P* = 0.0003), despite SpO_2_ alone showing a marginally negative association (*P* = 0.0603) and CRP maintaining a negative individual effect (*P* = 0.0491). Respiratory rate (Rrate) was positively associated with mean D-dimer (*P* = 0.0360), while its interaction with CRP (CRP × Rrate) showed a negative association (*P* = 0.0475), implying that D-dimer levels increase as the CRP × Rrate effect decreases.

Ct values were significantly and positively associated with mean D-dimer (*P* <0.0001), suggesting a link between viral load and inflammatory response. Mean D-dimer also showed a positive association with respiratory rate (*P* = 0.0360) but was indifferent to eGFR (*P* = 0.7127). The joint interaction of eGFR and respiratory rate (eGFR × Rrate) exhibited a negative partial association (*P* = 0.1107), indicating a potential rise in D-dimer levels when this interaction weakens.

Further, mean D-dimer was positively associated with the interaction of CRP and eGFR (CRP × eGFR) (*P* = 0.0007), although it remained negatively associated with CRP alone (*P* = 0.0491) and indifferent to both eGFR (*P* = 0.7127) and Cr (*P* = 0.3343). A negative partial association was observed with the interaction of eGFR and Cr (eGFR × Cr) (*P* = 0.1316), suggesting increased D-dimer levels when this interaction decreases. Overall, mean D-dimer was unaffected by the marginal effects of Cr and eGFR.

Regarding gender, mean D-dimer showed a partially negative association (*P* = 0.1669), implying that male patients may be more prone to elevated D-dimer levels and heightened inflammatory responses. Additionally, mean D-dimer was negatively associated with the duration of hospital stay (*P* = 0.0021), indicating elevated levels in patients staying for a shorter duration.

Turning to the variance model (Table 2), D-dimer variance was positively, though partially, associated with Ct values (*P* = 0.0934) and negatively related with the interaction of Ct and gender (Ct × Gen) (*P* = 0.0016), suggesting greater variability in D-dimer among male patients with lower Ct values. Variance also increased with higher SpO_2_ levels (*P* = 0.0081) and showed a strong positive association with CRP (*P* <0.0001), contrasting with its negative mean-level association.

The variance of D-dimer was positively and significantly associated with respiratory rate and negatively associated with hospital stay duration (*P* = 0.0014). It also showed a positive association with the interaction of Ct and duration (Ct × Dura) (*P* = 0.0036), indicating increased variability with stronger joint effects. Variance was negatively associated with urea (*P* = 0.2099) and the interaction of urea and CRP (urea × CRP) (*P* = 0.0048), suggesting increased variability as this interaction weakens. Similarly, the interaction of respiratory rate and CRP (Rrate × CRP) was negatively associated with variance (*P* = 0.0003), reinforcing the role of joint effects in modulating D-dimer variability.

Finally, variance was indifferent to eGFR (*P* = 0.8889) and showed a negative partial association with both urea (*P* = 0.2099) and the interaction of urea and eGFR (urea × eGFR) (*P* = 0.1669), indicating that reduced joint effects may contribute to increased variability in D-dimer levels.

### Response of CRP

The response variable CRP was modeled using JGLMs under both Gamma and Log-normal distributions (Table 3). Based on the AIC, the Gamma fit (AIC = 1290.736) was preferred over the Log-normal fit (AIC = 1307.272), indicating a better overall model performance.

**Table 3:** Results for mean and dispersion models for CRP from log-normal and gamma fit

Model	Covariates	Gamma fit				Log-normal fit			
		Estimate	S.E.	*t*	*P*-value	Estimate	S.E.	*t*	*P*-value
Mean	Constant	−11.9519	4.8155	−2.482	0.0144	−11.0012	5.2680	−2.088	0.0388
	Ct	0.4112	0.1362	3.020	0.0031	0.4031	0.1505	2.679	0.0084
	Urea	0.0033	0.0006	5.412	<0.0001	0.0030	0.0006	4.703	<0.0001
	Gen 2	0.3132	0.6996	0.448	0.6549	0.1878	0.7438	0.253	0.8007
	Rrate	0.1065	0.0155	6.866	<0.0001	0.1357	0.0169	8.006	<0.0001
	Rrate*Gen 2	−0.0659	0.0201	−3.283	0.0013	−0.0661	0.0215	−3.071	0.0026
	Dura	−0.3629	0.0776	−4.677	<0.0001	−0.4979	0.0878	−5.669	<0.0001
	SPO2	0.1359	0.0490	2.772	0.0064	0.1117	0.0547	2.040	0.0434
	Dura*SPO2	0.0038	0.0008	4.449	<0.0001	0.0053	0.0010	5.478	<0.0001
	DDimer	0.4883	0.0633	7.710	<0.0001	0.4861	0.0679	7.159	<0.0001
	SPO2*D-dimer	−0.0027	0.0005	−5.755	<0.0001	−0.0025	0.0005	−4.638	<0.0001
	Rrate*D-dimer	−0.0093	0.0010	−9.353	<0.0001	−0.0100	0.0010	−9.548	<0.0001
	eGFR	0.0304	0.0080	3.784	0.0002	0.0321	0.0093	3.441	0.0008
	Ct*eGFR	−0.0014	0.0003	−4.978	<0.0001	−0.0016	0.0003	−4.776	<0.0001
	age	0.0798	0.0236	3.376	0.0010	0.0769	0.0245	3.139	0.0021
	SPO2*age	−0.0009	0.0003	−3.612	0.0004	−0.0009	0.0003	−3.231	0.0016
	Cr	−0.1887	0.0395	−4.778	<0.0001	−0.1778	0.0417	−4.260	<0.0001
	Cr*Gen 2	0.1392	0.0440	3.163	0.0020	0.1407	0.0452	3.111	0.0023
	eGFR*Gen 2	0.0083	0.0039	2.128	0.0353	0.0089	0.0045	1.985	0.0493
	eGFR*Cr	−0.0168	0.0110	−1.526	0.1295	−0.0172	0.0125	−1.376	0.1712
	Ct*SPO2	−0.0033	0.0015	−2.256	0.0258	−0.0031	0.0016	−1.927	0.0562
	Urea*Gen 2	0.0016	0.0012	1.346	0.1807	0.0025	0.0013	1.998	0.0478
Dispersion	Constant	−1.99	4.05	−0.49	0.6250	−3.00	4.42	−0.68	0.4977
	age	−0.0458	0.0148	−3.09	0.0025	−0.0472	0.0154	−3.07	0.0026
	Ct	−0.1529	0.0558	−2.74	0.0070	−0.1730	0.0595	−2.91	0.0043
	Urea	0.1306	0.0347	3.76	0.0003	0.1465	0.0380	3.85	0.0002
	Rrate	0.0268	0.0377	0.71	0.4790	0.0539	0.0414	1.30	0.1959
	Cr	−0.581	0.192	−3.03	0.0030	−0.631	0.201	−3.14	0.0021
	Gen 2	0.075	0.367	0.20	0.8418	−0.132	0.394	−0.33	0.7419
	Cr*Gen 2	−0.234	0.168	−1.39	0.1669	−0.192	0.170	−1.13	0.2606
	Dura	−0.1836	0.0805	−2.28	0.0243	−0.1397	0.0781	−1.79	0.0758
	age*Dura	0.0030	0.0013	2.31	0.0225	0.0024	0.0013	1.82	0.0711
	Urea*Cr	0.0015	0.0006	2.46	0.0152	0.0020	0.0006	3.09	0.0025
	SPO2	0.0984	0.0344	2.86	0.0049	0.1185	0.0379	3.13	0.0022
	Urea*SPO2	−0.0011	0.0003	−3.61	0.0004	−0.0013	0.0003	−3.80	0.0002
	D-dimer	−0.1056	0.0338	−3.13	0.0022	−0.1080	0.0355	−3.04	0.0029
	D-dimer*Gen 2	0.1063	0.0352	3.02	0.0031	0.1136	0.0368	3.09	0.0025
	eGFR	−0.0596	0.0202	−2.95	0.0038	−0.0643	0.0216	−2.97	0.0036
	Ct*eGFR	0.0021	0.0007	2.91	0.0043	0.0022	0.0008	2.80	0.0059
	Urea*Rrate	−0.0013	0.0004	−3.41	0.0009	−0.0015	0.0004	−3.68	0.0003
AIC		1290.736				1307.272			

CRP: C-reactive protein; Ct value: cycle threshold value, eGFR: estimated glomerular filtration rate, SpO_2_: oxygen saturation, Dura: duration of hospital stay, Cr: creatinine, Rrate: respiratory rate, Gen: gender, AIC: Akaike information criterion.

**Figure 2:**
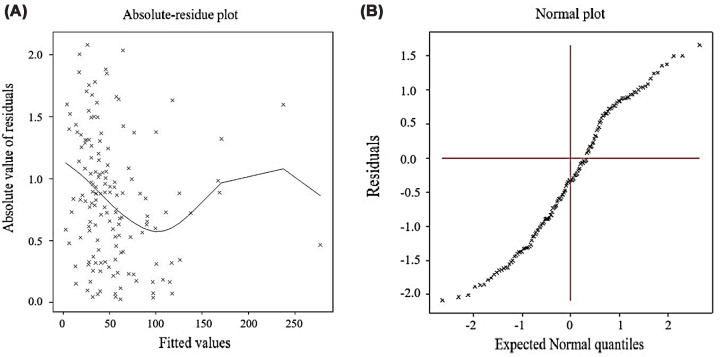
Diagnostic plots for joint gamma-fitted models of CRP For joint gamma-fitted models of CRP, (**A**) the absolute residuals plot with the fitted values, and (**B**) the normal probability plot for the mean model.

Absolute–residue plot displays the absolute values of residuals against the fitted values of the model (Figure 2A). The scattered ‘x’ marks represent individual data points, and the fitted line helps visualize the overall trend. The trend line appears relatively flat, suggesting that the residuals do not show increasing or decreasing variance with fitted values. This indicates that the model likely satisfies the assumption of constant variance, which supports the validity of the model’s error structure. Alternatively, a normal plot (Figure 2B) compares the distribution of residuals to a theoretical normal distribution. The ‘x’ marks represent observed residuals, and the cross lines help visualize symmetry around the origin. The residuals generally follow a linear pattern along the expected quantiles, with no major deviations or curvature. This suggests that the residuals are distributed quite normally, indicating a good model fit regarding distributional assumptions.

The mean CRP levels in COVID-19 patients demonstrated several significant associations with clinical and biochemical parameters. A positive correlation between mean CRP and age (*P* = 0.0010), oxygen saturation (SpO_2_) (*P* = 0.0064), and D-dimer levels (*P* <0.0001) was observed. CRP was also positively associated with respiratory rate (*P* <0.0001) and D-dimer concentrations, while showing a negative association with their joint interaction effect (Rrate × D-dimer) (*P* <0.0001), indicating that CRP levels increase as the combined influence of respiratory rate and D-dimer decreases.

Mean CRP was positively associated with Ct values (*P* = 0.0031) and eGFR (*P* = 0.0002), in contrast with its negative relation with serum Cr (*P* <0.0001). Gender solely was not found to affect CRP levels (*P* = 0.6549) significantly, but the interaction term Cr × Gen 2 (*P* = 0.0020) was positively associated, suggesting elevated CRP levels in female patients with higher Cr. Similarly, CRP was positively associated with serum urea, and although the interaction with gender was insignificant (*P* = 0.6549), a partial positive correlation was noted with Urea × Gen 2 (*P* = 0.1807), indicating higher CRP levels in females with elevated urea.

The joint effect of eGFR and gender (eGFR × Gen 2) was positively associated with CRP (*P* = 0.0353), suggesting higher CRP levels in female patients with better renal function. While CRP was positively associated with both Ct and SPO_2_, it revealed a negative correlation with their interaction (Ct × SpO_2_) (*P* = 0.0258), implying that CRP levels rise as their joint effect diminishes. Respiratory rate remained a strong positive predictor (*P* <0.0001), but its interaction with gender (Rrate × Gen 2) was negatively associated (*P* = 0.0013), indicating higher CRP levels in male patients with lower respiratory rates.

CRP levels were negatively associated with the duration of hospital stay (Dura) (*P* <0.0001) yet positively associated with the interaction of Dura and SpO_2_ (*P* <0.0001), suggesting increased CRP levels with higher combined effects of these variables. A negative association was also observed with the interaction of SpO_2_ and age (SPO_2_ × age) (*P* = 0.0004) and with the joint effect of Ct and eGFR (Ct × eGFR) (*P* <0.0001). Additionally, a negative correlation was found between CRP and the interaction of eGFR and Cr (eGFR × Cr) (*P* = 0.1295), indicating the CRP levels as the possible cause of increase as this joint effect declines.

Regarding variance, CRP variability was negatively correlated with age (*P* = 0.0025) and duration of hospital stay (*P* = 0.0243) but positively related with their interaction (age × Dura) (*P* = 0.0225), indicating greater variability with increasing combined age and hospitalization duration. Variance was positively associated with urea (*P* = 0.0003) and negatively with Cr (*P* = 0.0030), while their interaction (urea × Cr) showed a positive association (*P* = 0.0152), suggesting increased variability with stronger joint effects. CRP variance was also positively associated with SpO_2_ (*P* = 0.0049) and urea, but a negative correlation was found in their interaction (urea × SpO_2_) (*P* = 0.0004), indicating greater variability as the joint effect weakens. Finally, CRP variance was indifferent to gender (*P* = 0.8418) and partially negatively associated with Cr × Gen 2 (*P* = 0.1669), suggesting more pronounced variability among younger male patients.

### Cross-talk effect of CRP, D-dimer, and AKI

**Figure 3:**
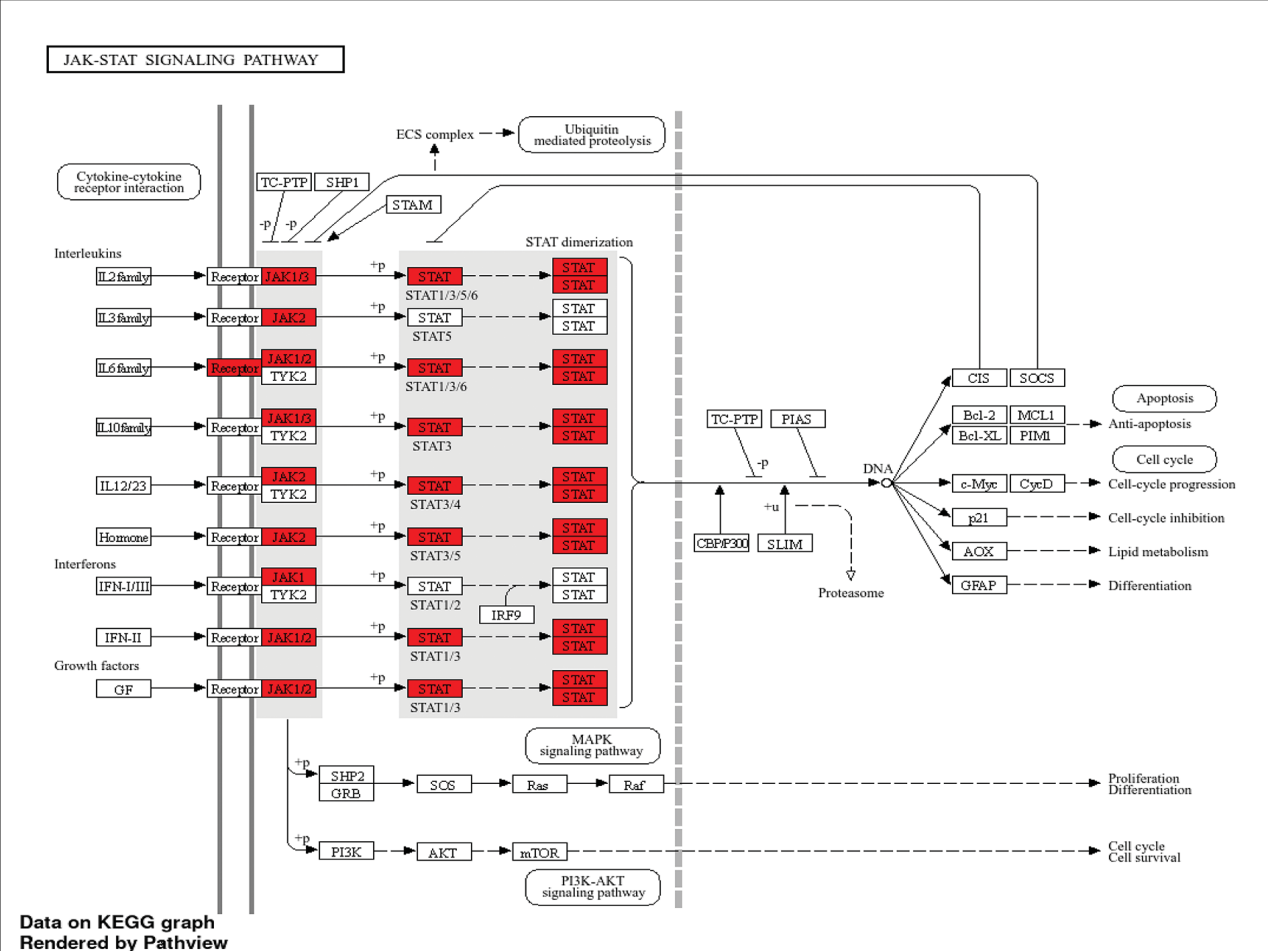
JAK/STAT signaling (hsa04630) IL-6/IL6R activates JAK1/JAK2-STAT3, inducing CRP and driving inflammatory and fibrotic kidney responses. Activation of JAK-STAT signaling by interleukins, interferons, and growth factors drives downstream outcomes including cell-cycle progression, anti-apoptosis, proliferation, differentiation, and lipid metabolism highlighting (red) indicating significantly up-regulated genes. JAK—Janus kinase; TYK2—tyrosine kinase 2; STAT—signal transducer and activator of transcription; IL—interleukin; IFN—interferon; GF—growth factor; SHP1—Src Homology region 2 domain-containing phosphatase-1; SHP2—Src Homology region 2 domain-containing phosphatase-2; GRB2—growth factor receptor-bound protein 2; Ras—rat sarcoma small GTPase; PI3K—phosphoinositide 3-kinase; AKT—protein kinase B; mTOR—mechanistic target of rapamycin; IRF9—Interferon regulatory factor 9; MAPK—mitogen-activated protein kinase; SH2—Src Homology 2 domain; SLIM—STAT-interacting LIM protein; Bcl-2—B-cell lymphoma 2; CycD—cyclin D; p21—cyclin-dependent kinase inhibitor 1A (CDKN1A); AOX—Acyl-CoA oxidase; GFAP—glial fibrillary acidic protein.

**Figure 4:**
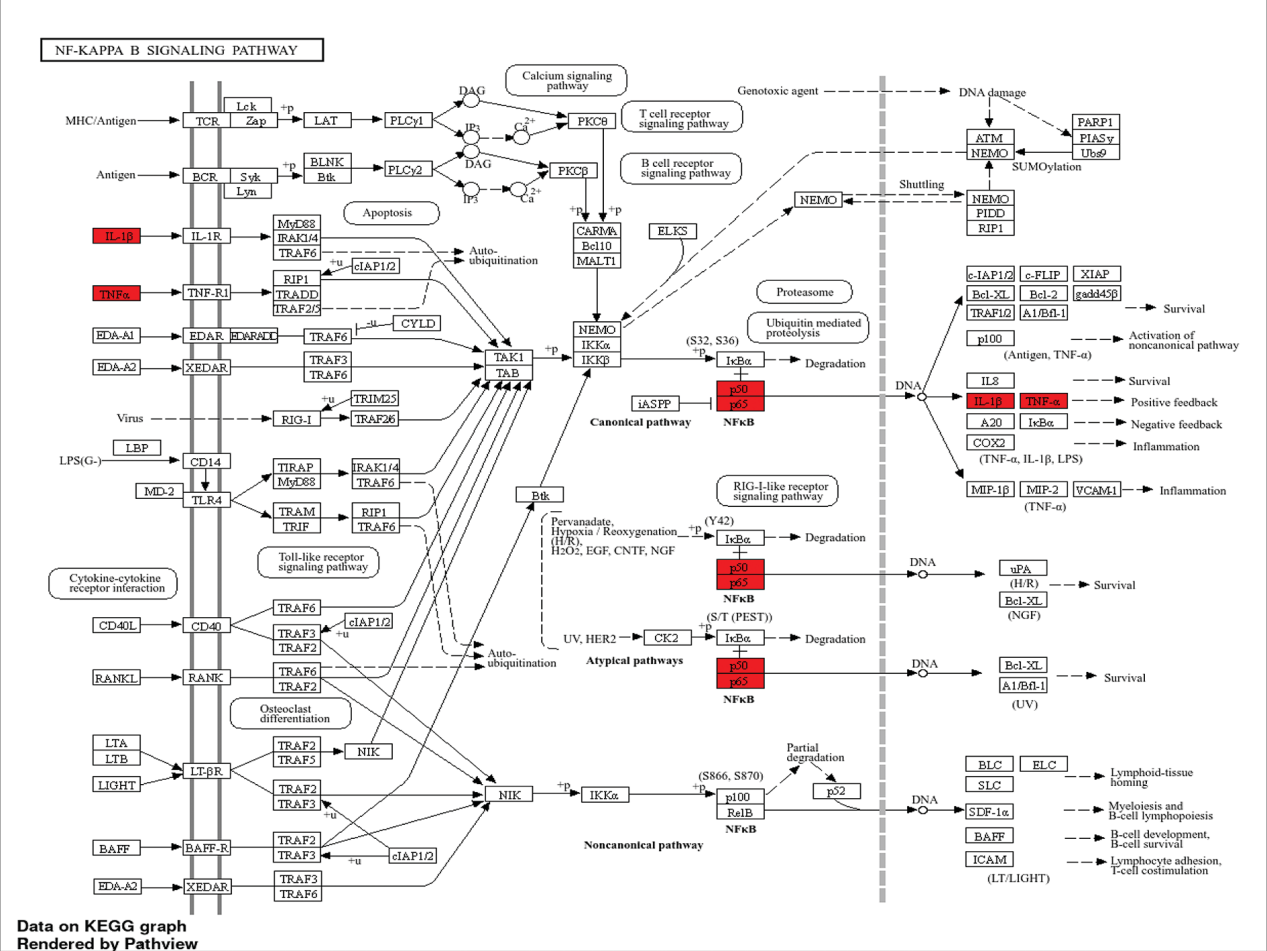
NF-κB signaling (hsa04064) Viral RNA and cytokines activate NF-κB (NFKB1/RELA), promoting TNF and IL1B expression and driving renal inflammation. Activation of NF-κB signaling through canonical, atypical, and noncanonical pathways drives pro-survival, inflammatory, and lymphoid development outcomes, with up-regulated genes (red) including IL-1β, TNFα, and NF-κB subunits reinforcing positive feedback loops promoting inflammation and cell survival. NF-κB—nuclear factor kappa-light-chain-enhancer of activated B cells; p50—NF-κB1 (p50 subunit); p65 (RelA)—v-Rel avian reticuloendotheliosis viral oncogene homolog A; RelB—RELB proto-oncogene; p52—NF-κB2 (processed subunit); p100—NF-κB2 precursor protein; TCR—T-cell receptor; BCR—B-cell receptor; IL-1β—interleukin-1 beta; IL-1R—interleukin-1 Receptor; TNF-α—tumor necrosis factor alpha; TNF-R1—tumor necrosis factor receptor 1; TLR4—Toll-like receptor 4; CD14—cluster of differentiation 14; CD40—cluster of differentiation 40; CD40L—CD40 ligand; RANK—receptor activator of NF-κB; MHC—major histocompatibility complex; LPS—lipopolysaccharide; LBP—lipopolysaccharide-binding protein; IKKα/β/γ—IκB kinase alpha/beta/gamma; IκBα—inhibitor of kappa B alpha; NIK—NF-κB-inducing kinase; PLCγ1/2—phospholipase C gamma 1/2; PKCβ/θ—protein kinase C beta/theta; DAG—diacylglycerol; IP3—inositol 1,4,5-trisphosphate; ATM—Ataxia telangiectasia-mutated kinase; PARP1—poly (ADP-ribose) polymerase 1; Ub—ubiquitin; CK2—casein kinase 2; IL-8—interleukin 8; A20—TNF alpha-induced Protein 3 (TNFAIP3); COX2—cyclooxygenase 2; MIP-2—macrophage inflammatory protein 2; VCAM1—vascular cell adhesion molecule 1; Bcl-2—B-cell lymphoma 2

**Figure 5:**
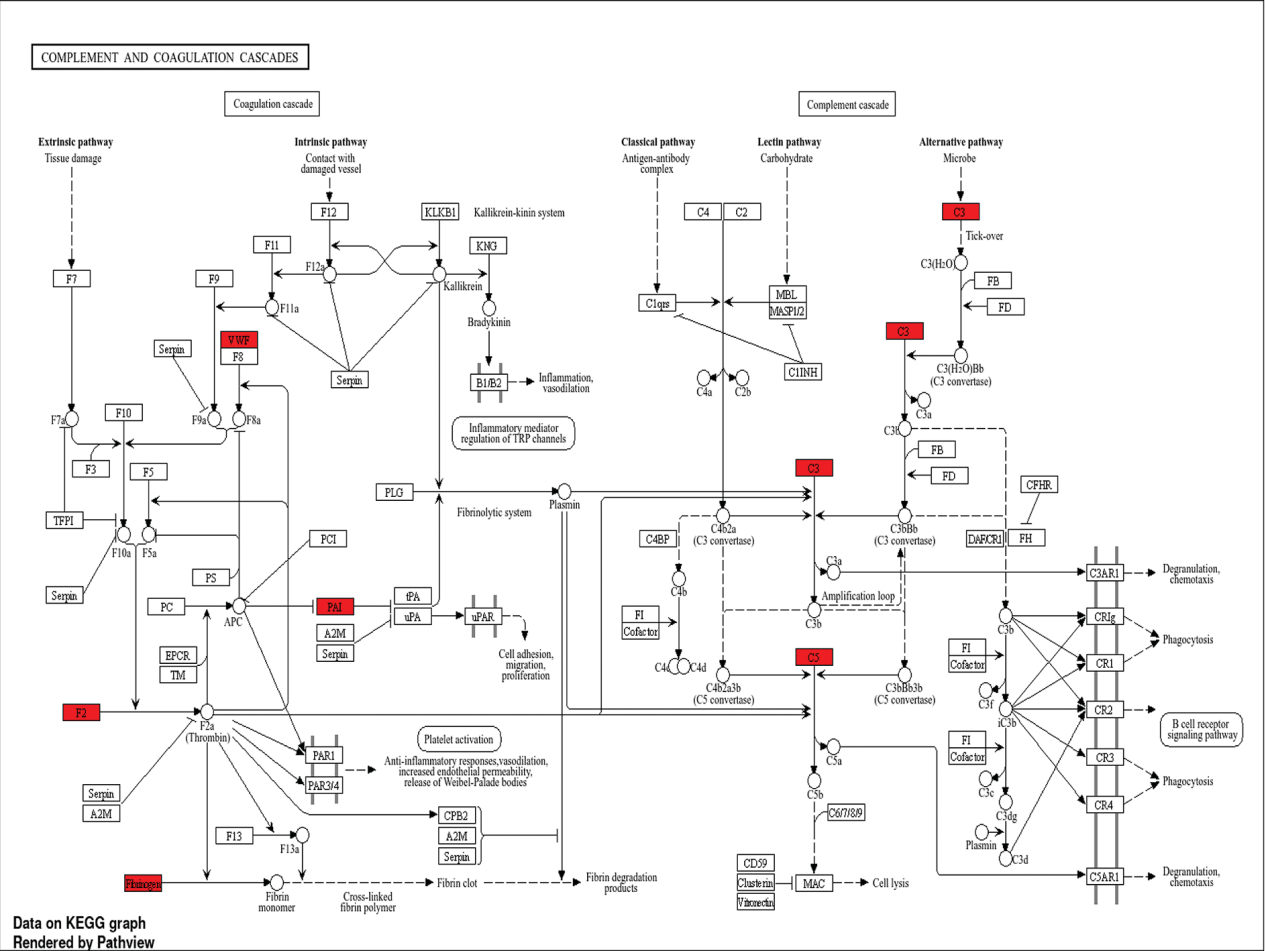
Complement and coagulation cascades (hsa04610) Activation of FGA/FGB/FGG, F2, VWF, C3, C5, and SERPINE1 promotes hypercoagulability, elevated D-dimer, microthrombosis, and complement-mediated renal injury. Up-regulated genes (red) converge on pro-coagulant, pro-inflammatory, and complement-activating outcomes, collectively promoting fibrin clot formation, cell lysis, phagocytosis, and degranulation. F2—coagulation factor II (prothrombin); F3—coagulation factor III (tissue factor); F5—coagulation factor V (Proaccelerin); F7—coagulation factor VII (proconvertin); F8—coagulation factor VIII (anti-hemophilic factor A); F9—coagulation factor IX (Christmas factor); F10—coagulation factor X (Stuart–Prower factor); F11—coagulation factor XI (plasma thromboplastin antecedent); F12—coagulation factor XII (hageman factor); F13—coagulation factor XIII (fibrin-stabilizing factor); PC—protein C; PS—protein S; APC—activated protein C; PCI—protein C inhibitor; EPCR—endothelial protein C receptor; TM—thrombomodulin; PAR1—protease-activated receptor 1; PAR3/4—protease-activated receptor 3/4; CPB2—carboxypeptidase B2 (thrombin-activatable fibrinolysis inhibitor); C1qrs—complement component 1q, 1r, and 1s complex; C4BP—C4b-binding protein; MBL—mannose-binding lectin; MASP1/2—MBL-associated serine protease 1/2; FB—complement factor B; FD—complement factor D; FH—complement factor H; CFHR—complement factor H-related protein; FI—complement factor I; CR1—complement receptor 1; CD59—cluster of differentiation 59 (protectin); MAC—membrane attack complex; ICAM—intercellular adhesion molecule; VCAM—vascular cell adhesion molecule.

**Figure 6:**
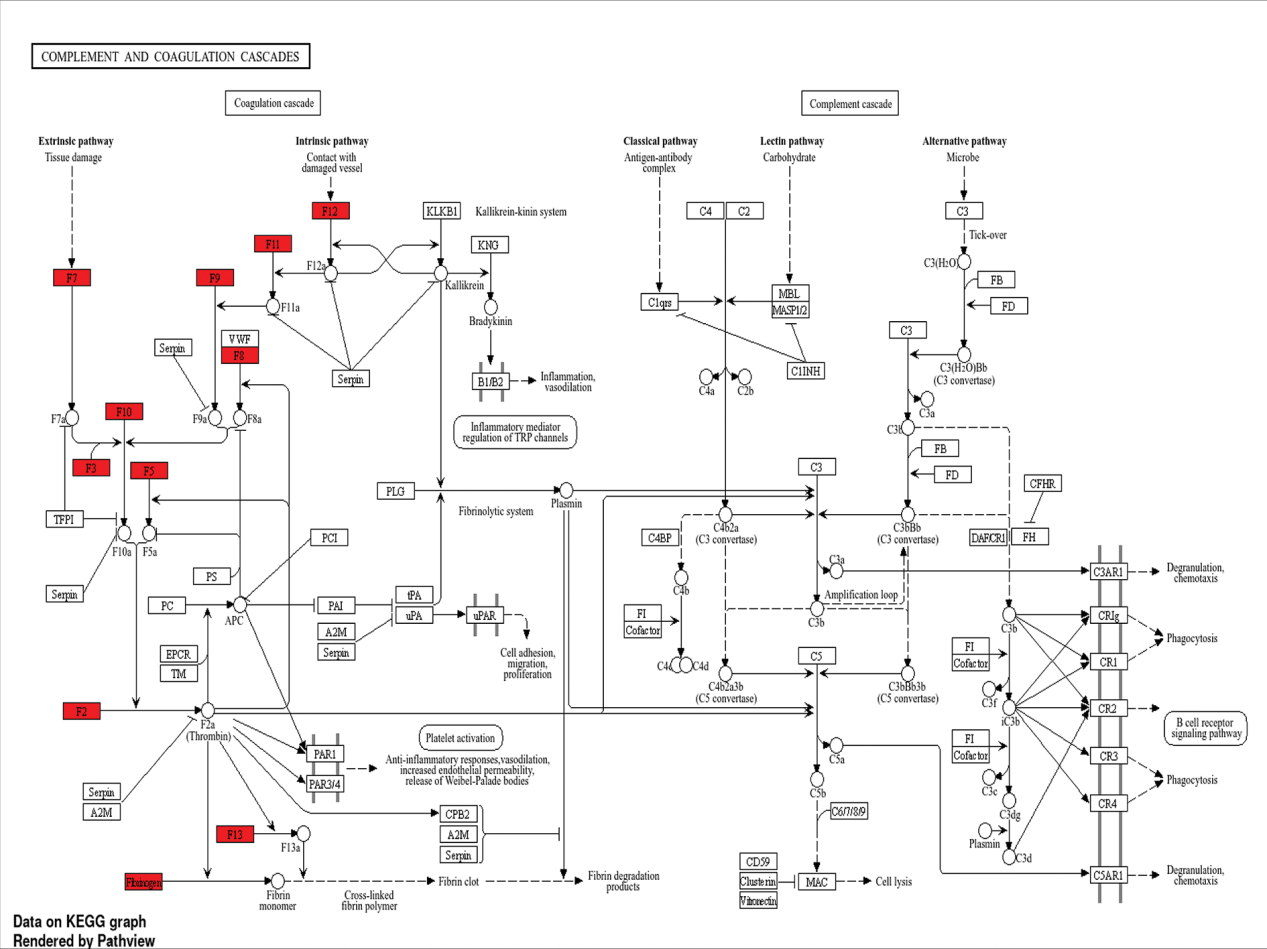
Complement and coagulation cascades (hsa04610) Activation of FGA/FGB/FGG, F2, VWF, C3, C5, and SERPINE1 promotes hypercoagulability, elevated D-dimer, microthrombosis, and complement-mediated renal injury. Broad up-regulation of coagulation factors (F2, F3, F5, F7, F9, F10, F11, F12, F13, VWF, and Fibrinogen) strongly activates (red) both intrinsic and extrinsic coagulation pathways, driving fibrin clot formation, platelet activation, and inflammation. F2—coagulation factor II (Prothrombin); F3—coagulation factor III (Tissue Factor); F5—coagulation factor V (Proaccelerin); F7—coagulation factor VII (Proconvertin); F8—coagulation factor VIII (anti-hemophilic Factor A); F9—coagulation factor IX (Christmas Factor); F10—coagulation factor X (Stuart–Prower factor); F11—coagulation factor XI (plasma thromboplastin antecedent); F12—coagulation factor XII (Hageman factor); F13—coagulation factor XIII (fibrin-stabilizing factor); PC—protein C; PS—protein S; APC—activated protein C; PCI—protein C inhibitor; EPCR—endothelial protein C receptor; TM—thrombomodulin; PAI—plasminogen activator inhibitor; PLG—plasminogen; PAR1—protease-activated receptor 1; PAR3/4—protease-activated receptor 3/4; KLKB1—plasma kallikrein (kallikrein B1); C1qrs—complement component 1q, 1r, and 1s complex; MBL—mannose-binding lectin; MASP1/2—MBL-associated serine protease 1/2; FB—complement factor B; CFHR—complement factor H-related protein; FI—complement factor I; DAF—decay-accelerating factor (CD55); CR1—complement receptor 1; CD59—cluster of differentiation 59 (Protectin); MAC—membrane attack complex.

COVID-19 is characterized by systemic inflammation, hypercoagulability, and endothelial dysfunction, all of which contribute to multi-organ injury, including AKI. Two of the most clinically relevant biomarkers—CRP and D-dimer—are directly linked to cytokine storm, coagulation dysregulation, and renal damage. Some common genes found are enlisted in Supplementary Tables S1–S3, which indicate their cross-talk effect (Figures 3-6 and Supplementary Figures S1–S3). The integrated analysis of inflammatory, coagulation, and signaling pathways highlights the multifactorial mechanisms by which COVID-19 contributes to kidney injury. The fourth table demonstrates the systemic inflammation and acute-phase protein induction mediated by IL-6, TNF, NF-κB, and STAT3 not only drive elevated CRP but also promote tubular apoptosis and fibrosis [19–23]. Complementing this, the fifth table underscores the role of coagulation and fibrinolysis imbalance, where excessive fibrin generation and impaired fibrinolysis sustain elevated D-dimer levels, leading to renal micro-thrombosis and ischemia [24–28]. Moreover, endothelial activation and complement cascade dysregulation aggravate glomerular injury, consistent with microvascular pathology observed in severe COVID-19 cases [26,27,29]. The KEGG/GO pathway mapping further integrates these findings, revealing convergence of cytokine signaling (IL-6/JAK-STAT, NF-κB), coagulation cascades, and hypoxia-driven HIF-1 responses that exacerbate tubular necrosis and promote long-term fibrotic remodelling through TGF-β signaling [23,30].

## Discussion

The analysis of D-dimer and CRP in relation to various biochemical and physiological parameters in COVID-19 patients provides in-depth information on their interconnected roles in inflammation, coagulation, organ dysfunction, and disease severity. The findings from both the mean and variance models of D-dimer and CRP highlight intricate interactions, reinforcing the importance of these biomarkers in COVID-19 management. Findings from both mean and variance models of D-dimer and CRP reveal nuanced relationships with renal and respiratory parameters, suggesting that elevated levels of these studied markers are not only indicative of hyperinflammatory states and thrombotic risk but also correlate with AKI and respiratory failure [31,32]. This reinforces their utility in predicting disease severity, guiding clinical decision-making, and tailoring therapeutic interventions.

By integrating these biomarkers into a unified framework, clinicians can better stratify risk, monitor progression, and anticipate complications—making D-dimer and CRP indispensable components of COVID-19 management when interpreted in conjunction with renal and respiratory indicators (as disease severity markers).

This comprehensive analysis highlights the intricate relationships between D-dimer and plasma CRP levels in COVID-19 patients and various clinical and biochemical parameters, with a precise emphasis on renal biomarkers. While serum Cr and BUN—which reflects serum urea concentration—are commonly used indicators of renal function, our study employed eGFR as a more sensitive measure. Unlike serum Cr alone, eGFR signifies patient age, gender, weight, and Cr levels, offering a more accurate assessment of renal functionality [33]. BUN, a nitrogenous waste product of protein catabolism, is unique in that it is both filtered by the glomerulus and reabsorbed in the renal tubules. Therefore, a progressive increase in BUN levels is indicative of renal failure, particularly in septic patients. Potential causes of elevated BUN in COVID-19 embrace acute tubular necrosis owing to sepsis and renal hypoperfusion, cytokine release syndrome, direct viral invasion of renal tubular cells, renal medullary hypoxia secondary to alveolar injury, and cardio-renal syndrome resulting from viral myocarditis [33].

The findings of our present study firmly declare that both CRP and D-dimer concentrations in SARS-CoV-2-infected individuals show a positive correlation with serum urea levels (BUN), while no profound relation was detected with serum Cr. Supporting our observations, Yin et al. [34] identified BUN as a predictive factor for 28-day mortality in COVID-19-positive individuals and recommended its routine monitoring. Casas Aparicio et al. [35] further demonstrated that BUN, rather than serum Cr, drives the progression of AKI in COVID-19, attributing the elevated BUN/Cr ratio to excessive BUN generation and increased tubular reabsorption. Similar deductions have been suggested by other researchers, reinforcing the predictive value of the BUN/Cr ratio for renal impairment in COVID-19. Therefore, based on our observations, it may be concluded that the BUN/Cr ratio is mainly increased by excessive BUN generation (catabolic state) combined with increased renal tubular reabsorption. Similar interpretations have also been suggested by many other researchers on how BUN/Cr can be used as a predictor for impaired renal functioning in other severe clinical conditions [36–38].

Cheng et al. [39] also stated significantly elevated serum titers of BUN and D-dimer in non-survivors compared with survivors, with ROC curve analysis depicting strong predictive power for in-hospital mortality: the area under the curve was 0.88 for BUN, 0.88 for D-dimer, and 0.87 for CRP. The blend of BUN and D-dimer improved predictive accuracy in comparison with any marker alone [39]. Furthermore, elevated BUN levels at admission were profoundly related to adverse outcomes, even after correcting for pre-existing renal disease [40]. Our study also revealed a significant positive correlation between CRP and eGFR, suggesting that glomerular function deteriorates proportionally with systemic inflammation. Hong et al. [41] similarly observed abnormally low eGFR during peak inflammatory phases of COVID-19, despite elevated BUN and Cr levels, and considered eGFR to be the more sensitive indicator.

Previously, Ebner et al. [42] reported a heterogeneous incidence of AKI in COVID-19 patients, ranging from 1% to 46%, while Chan et al. [43] found that mortality increased by 50% in patients with AKI compared with those without kidney involvement. Liu et al. [44] emphasized that dynamic changes in renal function parameters are more indicative of clinical deterioration than static values. However, the present investigation shows an absence of any significant relation between D-dimer concentration and serum Cr or eGFR. Similarly, Cheng et al. [39] did not consider serum Cr as a risk factor for unadvanced prognosis in COVID-19 patients.

Patients with a prehistory of renal disease are usually more vulnerable to SARS-CoV-2 infection, possibly because of their pro-inflammatory state and impaired immune function. In contrast, AKI during COVID-19 appears to be multifactorial, with hyperinflammation playing a central role. The progression of COVID-19 can be divided into three phases: early infection, pulmonary involvement, and severe hyperinflammation. The virus propagates into the host cells through ACE2 receptors of the renin-angiotensin-aldosterone system, and in individuals with CKD, systemic up-regulation of ACE2 may facilitate viral entry. Renal impairment in severe COVID-19 may also arise as a consequence of direct cytopathic effects, as autopsies have shown evidence of brush border sloughing, hyaline casts, microthrombi, and interstitial fibrosis—hallmarks of acute tubular necrosis [45]. Additionally, cytokine-mediated damage plays a major role, with the cytokine storm leading to uncontrolled pulmonary inflammation, acute respiratory distress syndrome, and increased mortality. During acute illness, unregulated viral replication triggers profound epithelial and endothelial cell death and vascular leakage, triggering innate and adaptive immune responses and the release of pro-inflammatory cytokines and chemokines. Such cytokines can trigger apoptotic changes in the epithelial cells of renal tubules, while SARS-CoV-2-mediated ACE2 down-regulation, shedding, and antibody-dependent enhancement further exacerbate inflammation [46,47].

Additionally, while mean D-dimer showed partial negative associations with gender (*P* = 0.1669), implying a higher predisposition for hypercoagulability in males, CRP revealed non-significant marginal associations with gender (*P* = 0.6549). Nonetheless, the positive interactions between Cr and gender (*P* = 0.0020) for CRP levels and negative associations between respiratory rate and gender interactions (*P* = 0.0013) suggest that inflammatory and coagulative responses may differ subtly between male and female patients. Such findings corroborate with existing evidence of sex-based differences in immunomodulatory responses to COVID-19 [48].

Also, the positive association of D-dimer with viral load, as indicated by Ct values (*P* <0.0001), underscores the impact of active viral replication on inflammatory and coagulative states. Variance analysis revealed a positive association with SpO_2_ (*P* = 0.0081), suggesting fluctuating D-dimer levels as oxygenation improves, while the negative association with duration of hospital stay (*P* = 0.0014) reflected stabilization of coagulation markers over time.

Furthermore, CRP, a key acute-phase reactant, exhibited positive associations with multiple parameters, including age (*P* = 0.0010), oxygen saturation (SpO_2_) (*P* = 0.0064), and D-dimer (*P* <0.0001). The strong association between CRP and D-dimer highlights the interplay between inflammation and coagulation cascades in COVID-19. Elevated CRP levels in patients with high urea concentrations and joint effects of urea and gender (*P* = 0.1807) reinforce the role of renal dysfunction in exacerbating systemic inflammation, particularly in females.

Moreover, cross-talk pathway analysis (using GO KEGG) of CRP, D-dimer, and kidney injury condition provided mechanistic evidence suggesting that the interplay between inflammation, coagulopathy, and cellular stress responses underlies the progression from acute injury to CKD in COVID-19 patients.

Renal function emerges as a critical determinant of both coagulative and inflammatory states, underscoring the need for integrated renal monitoring in severe COVID-19 cases. Additionally, the significant association of D-dimer with viral load highlights the importance of early viral load suppression to mitigate downstream complications.

Gender-specific findings warrant further investigation to tailor treatment strategies and ensure equitable care. Furthermore, variability analyses suggest that fluctuating inflammatory and coagulation responses over the disease course should be considered when designing therapeutic interventions.

## Conclusion

The combined analysis of D-dimer and CRP models elucidates their pivotal roles in the inflammatory and coagulative landscapes of COVID-19. Elevated levels of these markers are known to be associated with disease severity, but their combined effects with other parameters like urea, Cr, and SpO_2_ provide additional layers of understanding regarding the pathogenesis. In addition, monitoring interaction effects, rather than relying solely on individual marker levels, may offer a more comprehensive approach for risk stratification and management. By integrating mean and variance models, the present study presents a commendable framework for understanding the interplay between inflammation, coagulation, and organ dysfunction, paving the way for more personalized and effective patient management strategies.

## Limitations

Despite the fact that the present study provides valuable insights, its retrospective nature and dependence on secondary data may have limitations related to confounding factors and generalizability. Future prospective studies with larger, diverse cohorts are required for the validation of these findings. Mechanistic studies investigating the molecular pathways underlying observed associations would further enhance our understanding regarding the progression of pathogenesis and will help in developing strategies for specific therapeutic targeting.

## Supplementary Material

Supplementary Figures and Tables S1-S3

## Data Availability

The data supporting the present study’s findings can be obtained from the corresponding author upon reasonable request.

## Abbreviations

ACE2: angiotensin-converting enzyme 2
AIC: akaike information criterion
AKI: acute kidney injury
BUN: blood urea nitrogen
CKD: chronic kidney disease
Cr: creatinine
CRP: C-reactive protein
Ct: cycle threshold
eGFR: estimated glomerular filtration rate
IQR: inter-quartile range
JGLM: joint generalized linear model
PTC: proximal tubular cells
SARS-CoV-2: severe acute respiratory syndrome coronavirus 2
WHO: World Health Organization
