# Supplementary material for: Renal function dynamics in COVID-19: exploring biomarker interactions with D-dimer and C-reactive proteins
Source: Biosci Rep. 2026 Apr 22;46(4):BSR20254002. doi: 10.1042/BSR20254002 (PMC13136562; doi:10.1042/BSR20254002)
Supplement: Supplementary Figures and Tables S1-S3 [file BSR-2025-4002_supp.pdf]

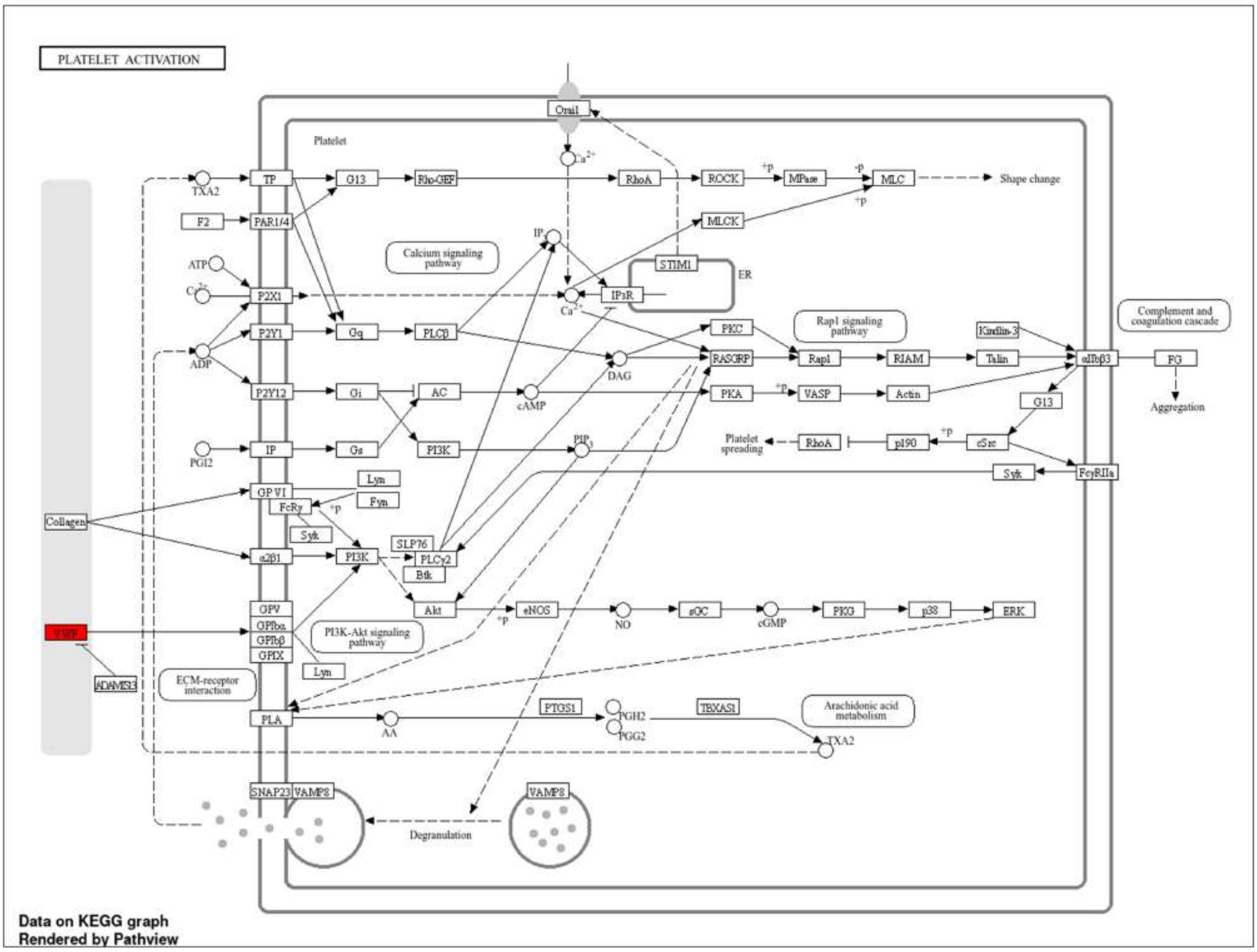

Supplementary Figure 1: Platelet activation (hsa04611): Endothelial activation via VWF, ICAM1, VCAM1, and SELE promotes thrombosis, leading to capillary plugging, proteinuria, and reduced eGFR. Upregulation of VWF (red) facilitates platelet adhesion and activation, converging on downstream outcomes of platelet aggregation, shape change, spreading, and degranulation, with crosstalk to the complement and coagulation cascades. TXA2 – Thromboxane A2; TP – Thromboxane Prostanoid Receptor; F2 – Coagulation Factor II (Prothrombin); PAR1/4 – Protease-Activated Receptor 1/4; ATP – Adenosine Triphosphate; ADP – Adenosine Diphosphate; AC – Adenylyl Cyclase; cAMP – Cyclic Adenosine Monophosphate; PI3K – Phosphoinositide 3-Kinase; PLCβ – Phospholipase C beta; PLCγ2 – Phospholipase C gamma 2; PIP2 – Phosphatidylinositol 4,5-bisphosphate; PIP3 – Phosphatidylinositol 3,4,5-trisphosphate; DAG – Diacylglycerol; IP3 – Inositol 1,4,5-trisphosphate; RIAM – Rap1-GTP-Interacting Adaptor Molecule; Talin – Cytoskeletal Protein Talin; Kindlin-3 – Integrin-Binding Protein Kindlin-3; VASP – Vasodilator-Stimulated Phosphoprotein; Actin – Actin Cytoskeletal Protein; Btk – Bruton's Tyrosine Kinase; Akt – Protein Kinase B; eNOS – Endothelial Nitric Oxide Synthase; NO – Nitric Oxide; cGMP – Cyclic Guanosine Monophosphate; PKG – Protein Kinase G; p38 – p38 Mitogen-Activated Protein Kinase; ERK – Extracellular Signal-Regulated Kinase; PLA – Phospholipase A; AA – Arachidonic Acid; PTGS1 – Prostaglandin-Endoperoxide Synthase 1 (Cyclooxygenase-1)

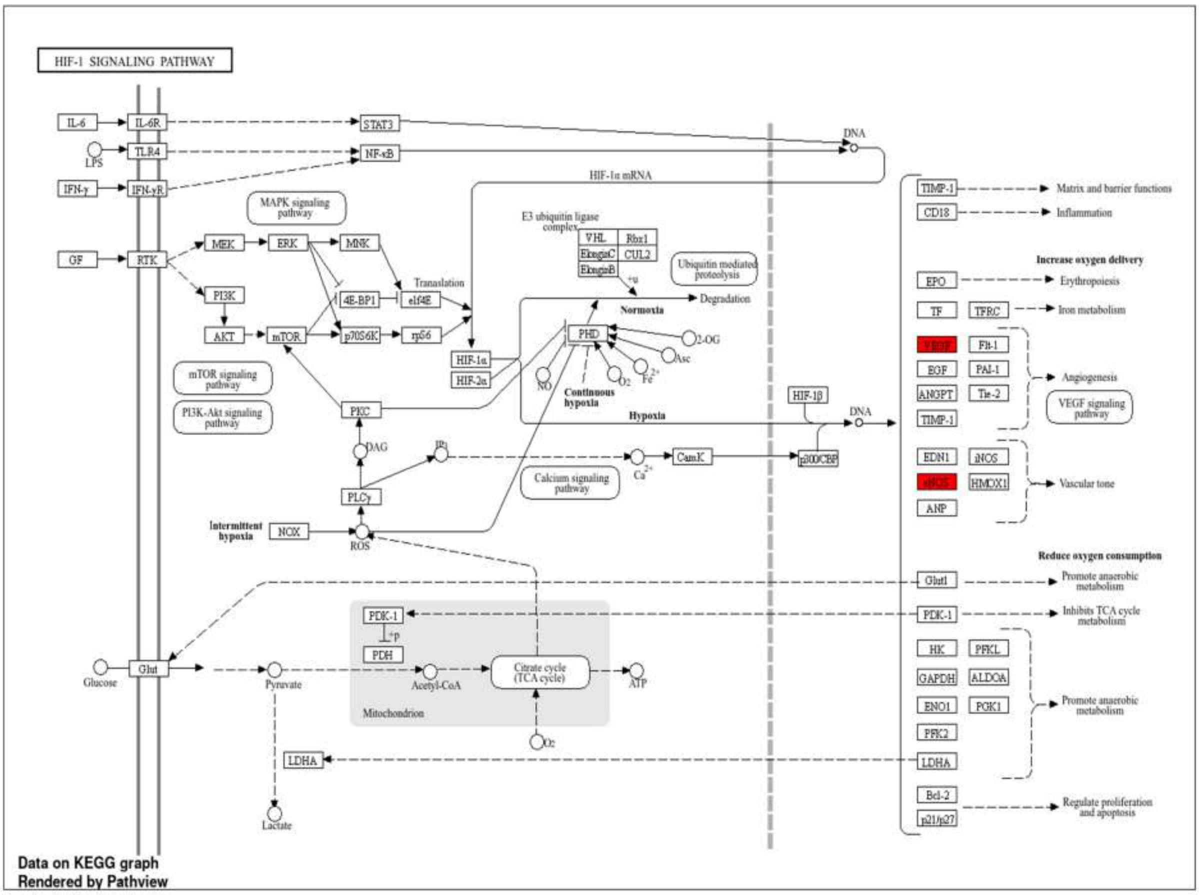

Supplementary Figure 2: HIF-1 signaling (hsa04066): Hypoxia-induced HIF1A activation (from lung injury) exacerbates renal ischemia and tubular necrosis. Upregulation of VEGF and eNOS (red) under hypoxic HIF-1 activation drives angiogenesis and vascular tone modulation, while promoting anaerobic metabolism and suppressing TCA cycle activity to reduce oxygen consumption. IL-6 – Interleukin-6; IL-6R – Interleukin-6 Receptor; LPS – Lipopolysaccharide; TLR4 – Toll-Like Receptor 4; IFN-γ – Interferon Gamma; IFN-γR – Interferon Gamma Receptor; GF – Growth Factor; RTK – Receptor Tyrosine Kinase; MAPK – Mitogen-Activated Protein Kinase; MEK – Mitogen-Activated Protein Kinase Kinase; ERK – Extracellular Signal-Regulated Kinase; MNK – MAP Kinase-Interacting Serine/Threonine Kinase; PI3K – Phosphoinositide 3-Kinase; AKT – Protein Kinase B; mTOR – Mechanistic Target of Rapamycin; PKC – Protein Kinase C; DAG – Diacylglycerol; IP3 – Inositol Trisphosphate; PLCγ – Phospholipase C Gamma; NOX – NADPH Oxidase; ROS – Reactive Oxygen Species; STAT3 – Signal Transducer and Activator of Transcription 3; NF-κB – Nuclear Factor Kappa B; HIF-1α – Hypoxia-Inducible Factor 1 Alpha; Glut – Glucose Transporter; PDK-1 – Pyruvate Dehydrogenase Kinase 1; PDH – Pyruvate Dehydrogenase; CD18 – Cluster of Differentiation 18 (Integrin Beta-2); VEGF – Vascular Endothelial Growth Factor; Flt-1 – FMS-Like Tyrosine Kinase 1 (VEGFR-1); EGF – Epidermal Growth Factor; iNOS – Inducible Nitric Oxide Synthase; eNOS – Endothelial Nitric Oxide Synthase; PGK1 – Phosphoglycerate Kinase 1; PFK2 – Phosphofructokinase-2; Bcl-2 – B-Cell Lymphoma 2

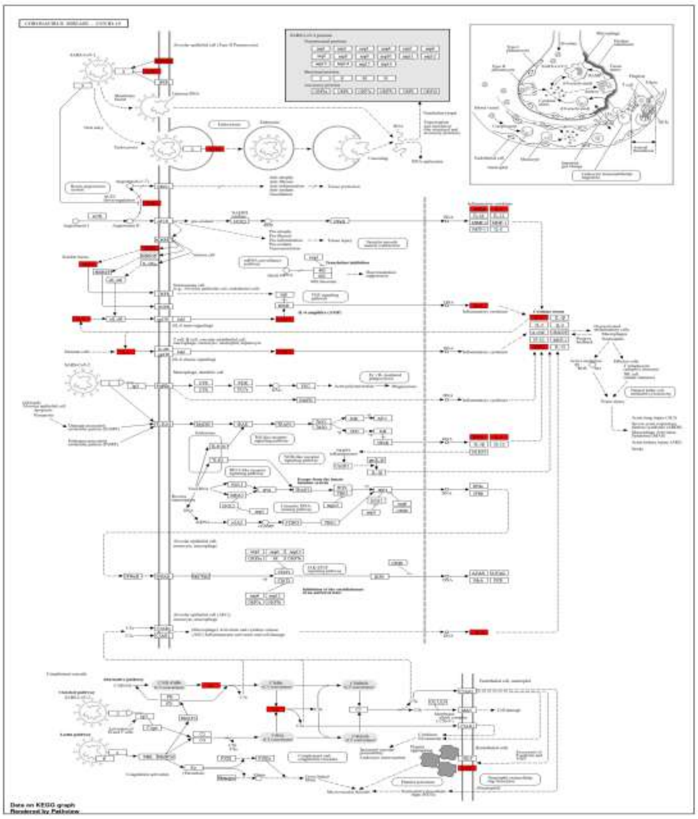

Supplementary Figure 3: COVID-19 pathway (hsa05171): ACE2/TMPRSS2 mediate viral entry, while IL6, TNF, STAT3, VWF, and C3 drive inflammation and coagulopathy, linking infection to kidney injury. Widespread upregulation (red) of inflammatory mediators (IL-1β, IL-6, IL-12, MMP genes, complement components and Fibrinogen) drives cytokine storm, complement activation, coagulation, and NETosis, culminating in acute lung injury, ARDS, and multi-organ damage. SARS-CoV-2 – Severe Acute Respiratory Syndrome Coronavirus 2; S – Spike Protein; ACE2 – Angiotensin-Converting Enzyme 2; HRP2 – Histidine-Rich Protein 2; MAS1 – MAS1 Proto-Oncogene G Protein-Coupled Receptor; ACE – Angiotensin-Converting Enzyme; AT1R – Angiotensin II Type 1 Receptor; IL-6Rα – Interleukin-6 Receptor Alpha; Jak1 – Janus Kinase 1; SYK – Spleen Associated Tyrosine Kinase; PI3K – Phosphoinositide 3-Kinase; PLC2 – Phospholipase C 2; DAG – Diacylglycerol; FCC – Fragment Crystallizable Region; MAPK – Mitogen-Activated Protein Kinase; MnSOD – Manganese Superoxide Dismutase; Tab1 – TGF-Beta Activated Kinase 1 Binding Protein 1; Tab2 – TGF-Beta Activated Kinase 1 Binding Protein 2; IKK – IκB Kinase; IκB – Inhibitor of Kappa B; NF-κB – Nuclear Factor Kappa B; AP-1 – Activator Protein 1; TLR7/8 – Toll-Like Receptor 7/8; TLR2 – Toll-Like Receptor 2; NLRP3 – NLR Family Pyrin Domain Containing 3; CASP1 – Caspase-1; cGAS – Cyclic GMP-AMP Synthase; cGAMP – Cyclic Guanosine Monophosphate–Adenosine Monophosphate; STING – Stimulator of Interferon Genes; IFNα – Interferon Alpha; IFNβ – Interferon Beta; IFNα/β – Interferon Alpha/Beta; IFNAR – Interferon Alpha/Beta Receptor; STAT1 – Signal Transducer and Activator of Transcription 1; STAT2 – Signal Transducer and Activator of Transcription 2; TYK2 – Tyrosine Kinase 2; PKR – Protein Kinase R; C2AR1 – Complement Component 2a Receptor 1; MASP – MBL-Associated Serine Protease; MBL – Mannose-Binding Lectin; C2 – Complement Component 2; ROS – Reactive Oxygen Species; NF-κB – Nuclear Factor Kappa B; IL-1β – Interleukin-1 Beta; ALI – Acute Lung Injury; ARDS – Acute Respiratory Distress Syndrome; MAS – Macrophage Activation Syndrome; AKI – Acute Kidney Injury; TLRP – Toll-Like Receptor Protein; IFN2α – Interferon 2 Alpha; IFN2β – Interferon 2 Beta; Angiotensin (1–7) – Angiotensin 1–7 Peptide

Supplementary Table 1: Genes driving CRP and D-dimer elevation in COVID-19

Biomarker	Gene Category	Key Genes	Regulation in COVID-19	Mechanism / Effect	References
**CRP (C-reactive protein)**	Output protein	CRP	Upregulated	Liver cells increase CRP synthesis in response to IL-6 signalling.	[5, 21-22]
	Upstream cytokines	IL6, IL1B, TNF	Upregulated	SARS-CoV-2 triggers immune cells → cytokine storm → ↑ IL-6, IL-1β, TNF.	[21-22]
	Cytokine receptor & signalling	IL6R, JAK1, JAK2, STAT3	Activated (↑)	IL-6 binds IL6R → JAK/STAT3 pathway activates CRP gene transcription.	[21-22]
	Transcription factors	NFKB1, RELA	Upregulated	NF-κB activated by viral infection → enhances CRP & other inflammatory gene expression.	[22-23]
	Other acute-phase proteins	SAA1, SAA2, HP	Upregulated	Co-expressed with CRP as part of acute-phase response.	[5, 21]
**D-dimer (fibrin degradation product)**	Fibrinogen (clot precursor)	FGA, FGB, FGG	Upregulated	Liver produces more fibrinogen under IL-6 drive → more fibrin clots → ↑ D-dimer.	[21, 24]
	Coagulation cascade (clotting factors)	F2, F3, F5, F7, F8, F9, F10, F11, F12, F13A1, F13B	Upregulated	Cytokines & endothelial injury → increased coagulation factor activity → hypercoagulation.	[24-25]
	Fibrinolysis (clot breakdown)	PLG, PLAU, PLAT	Activated (↑ activity)	Plasmin system breaks down fibrin → generates D-dimer.	[24]
	Inhibitors or regulators	SERPINE1 (PAI-1)	Upregulated	Inhibits fibrinolysis → clots persist longer (paradox: still more breakdown products).	[24-25]
		SERPINF2 (α2-antiplasmin)	Upregulated	Blocks plasmin → reduced fibrinolysis efficiency.	[24]
		PROC, PROS1, SERPINC1, THBD	Downregulated	Natural anticoagulants suppressed → favors thrombosis → ↑ D-dimer.	[24-25]
	Platelet or endothelial activation	VWF	Upregulated	Endothelial damage → ↑ von Willebrand factor → platelet adhesion & clotting.	[25-27]
		ICAM1, VCAM1, SELE	Upregulated	Endothelial activation → inflammation & microthrombosis.	[26-28]

Supplementary Table 2: Pathways linking CRP/D-dimer elevation to kidney damage in COVID-19

Pathway	Key Upregulated Genes	Effect on CRP / D-dimer	How This Damages Kidney	References
**Inflammation (CRP axis)**	IL6, IL1B, TNF	↑ Cytokines stimulate CRP production	Cytokine storm → tubular injury, apoptosis, fibrosis	[21-22]
	IL6R, JAK1, JAK2, STAT3	↑ Signal transduction → CRP ↑	Prolonged STAT3 signaling → renal inflammation	[21-22]
	NFKB1, RELA	↑ NF-κB activation → CRP ↑	NF-κB induces inflammatory genes → renal cell damage	[21-23]
**Acute-phase proteins**	CRP, SAA1, SAA2, HP	Direct ↑ of CRP & co-proteins	CRP deposition in glomeruli, complement activation	[21, 29]
**Coagulation (D-dimer axis)**	FGA, FGB, FGG	↑ Fibrinogen → more fibrin → ↑ D-dimer	Fibrin deposits in glomeruli → ischemia	[24, 29]
	F2 (Thrombin), F3 (Tissue factor), F5, F7, F8, F9, F10, F11, F12, F13A1, F13B	↑ Coagulation cascade → clot formation	Microthrombosis in renal capillaries → AKI	[24-25]
**Fibrinolysis (clot breakdown)**	PLG, PLAU, PLAT	↑ Breakdown of fibrin → ↑ D-dimer	Ongoing clot turnover stresses renal microvasculature	[24]
**Inhibitors (pro-thrombotic shift)**	SERPINE1 (PAI-1), SERPINF2 (α2-antiplasmin)	↑ Inhibit fibrinolysis (clots persist)	Reduced blood flow, renal ischemia	[24-25]
**Endothelial injury**	VWF, ICAM1, VCAM1, SELE	↑ Endothelial activation → more clotting & inflammation	Microvascular injury, proteinuria, reduced eGFR	[26-28]
**Complement activation**	C3, C5	↑ Complement activation enhances clotting	Complement-mediated glomerular injury	[25, 28]

Supplementary Table 3: Shared KEGG/GO pathways linking COVID-19 and Acute Kidney Injury (AKI)

Pathway (KEGG/GO)	Key Shared Genes	Role in COVID-19	Role in AKI	References
**Cytokine–cytokine receptor interaction (hsa04060)**	IL6, IL1B, TNF	Cytokine storm → systemic inflammation	Promotes tubular inflammation, apoptosis	[5, 21-22]
**JAK/STAT signalling (hsa04630)**	IL6R, JAK1, JAK2, STAT3	IL-6 activates CRP production	Drives inflammatory + fibrotic responses in kidney	[21-22]
**NF-κB signalling (hsa04064)**	NFKB1, RELA, TNF, IL1B	Viral RNA & cytokines activate NF-κB	NF-κB drives renal inflammatory gene expression	[5, 22-23]
**Acute phase response (IL-17/Inflammatory axis)**	CRP, SAA1, SAA2, HP	CRP ↑ with IL-6 drive	CRP & SAA deposition aggravates glomerular injury	[21, 29]
**Complement & coagulation cascades (hsa04610)**	FGA, FGB, FGG, F2, VWF, C3, C5, SERPINE1	Hypercoagulability, ↑ D-dimer	Microthrombosis, ischemic AKI, complement-mediated glomerular injury	[24-25, 28]
**Platelet activation (hsa04611)**	VWF, ICAM1, VCAM1, SELE	Endothelial activation → thrombosis	Capillary plugging, proteinuria, reduced eGFR	[26-27]
**HIF-1 signalling (hsa04066)**	HIF1A	Hypoxia from lung injury	Worsens renal ischemia and tubular necrosis	[30]
**TGF-β signalling (hsa04350)**	TGFB1, SMADs	Tissue remodelling or fibrosis post-COVID	Renal fibrosis → risk of CKD after AKI	[23, 29]
**COVID-19 pathway (hsa05171)**	ACE2, TMPRSS2, IL6, TNF, STAT3, VWF, C3	Explains viral entry + inflammation + coagulopathy	Integrates viral effects with kidney injury	[5, 21, 23]
**Apoptosis / p53 signalling**	TP53, BAX, CASP3	Viral stress induces apoptosis	Tubular epithelial cell apoptosis in AKI	[23, 29]
